# *Preventing Chronic Disease *in 2023: More Volunteers, New Appointments, Upcoming Collections, Acknowledgment of Guest Editorial Board on Racism, and Updates on Diversity, Equity, and Inclusion Initiatives

**DOI:** 10.5888/pcd20.230131

**Published:** 2023-06-29

**Authors:** Leonard Jack

**Affiliations:** 1 Preventing Chronic Disease, Office of Medicine and Science, National Center for Chronic Disease Prevention and Health Promotion, Centers for Disease Control and Prevention, Atlanta, Georgia

This year the motto for *Preventing Chronic Disease* (PCD) ought to be, “More of everything in 2023!” I’m pleased and proud to share the journal’s accomplishments to date and preview what we expect in the coming months. 

This has been a year of change and growth for the journal. While we continue to focus on content that aligns with our vision to disseminate peer-reviewed public health findings, innovations, and practices, we have also committed to publishing more collections in 2023 than ever before. The decision to publish up to 7 collections this year — almost twice as many as in 2022 — is due in part to our commitment to best practices related to health equity and to diversity, equity, and inclusion. This commitment is reflected not only in the topics that we will publish this year, but also in the makeup of the most recent appointments to our volunteer boards.

We also seek to provide more detailed guidance to authors on addressing issues of health equity in preparing manuscripts. We have posted a series of questions on the PCD website that address health equity considerations both in the process of research and in manuscript preparation. In presenting these guidelines, PCD joins with other journals in the forefront of the scholarly publishing community to frankly and directly address issues such as racism, negative social determinants of health, and other health inequities, looking to solutions that ameliorate these conditions and substantively and equitably improve the health and well-being of everyone.

## Volunteer Appointments

PCD has an impressive group of volunteers who serve as associate editors and on our Editorial Board and our Statistics Review Committee. We are pleased to continue to recruit such volunteers who bring a wealth of training, expertise, and experience to the journal, providing hundreds of hours of volunteer service. So far this year, we have appointed more than 20 new volunteers. These individuals come from a diverse background and build on the journal’s knowledge base in existing and new areas. Their fields of expertise are varied and include geographic information systems, statistical modeling, program evaluation, sleep health, health equity, racism and health, multiple chronic diseases, psychological health and chronic disease, data modernization, and more.

As part of this volunteer recruitment effort, the journal has launched a new initiative involving students. Considerable discussion has arisen regarding the need to increase the diverse pool of people in scholarly publishing to serve not only as authors but also as peer reviewers and as volunteers on boards and in leadership operational roles. To this end, we are pleased to announce formation of our inaugural Student Scientific Writing and Review Training Committee. This committee will consist of 10 students, 2 each at the high school, undergraduate, master’s, doctoral, and postdoctoral levels. Committee members will have an opportunity to learn more about effective scientific writing, including how to conduct peer review. They also will serve as student advisors to the journal and work closely with members of one or more of the journal’s volunteer boards.

## Upcoming Collections

The journal’s international reach allows us to publish content that addresses a range of timely public health concerns, and this year we have maintained and even expanded our commitment to identify topic areas that advance research, evaluation, practice, and public health policies to improve population health. We encourage interested authors to submit inquiries (PCDeditor@cdc.gov) to determine whether a manuscript’s topic area is of interest to the journal. We released several calls for papers on topics the journal has not addressed in past collections, recognizing the need to focus increased attention on several important and timely issues in public health.

Our first collection of the year, featuring 6 articles, is entitled *Combating Racism Through Research, Training, Practice, and Public Health Policies*. In April 2021, CDC declared racism a public health threat, identifying it as one of the fundamental drivers of health inequities (2). Racism, a root cause of health inequalities, is detrimental to health and is a significant source of stress across the lifespan (3). Racism has created and sustained common factors that contribute to health inequities and remains a worldwide public health challenge that requires open discussion. The collection will include peer-reviewed articles that offer insight into the role public health can play in combating racism through research, training, practice, and public health policies. This collection will also discuss future research, training, and policy solutions for combating racism and understanding its negative effects on health. We thank the members of the collection’s Guest Editorial Board for their contributions and feedback in the development of the collection’s Call for Papers, peer review of submissions, and input on topic areas of interest, which continue to advance research, evaluation, and practice on the effects of racism on health and on multipronged approaches to intervene and improve population health (Appendix). 

PCD’s second collection of 2023 is entitled *Health Equity in Action: Research, Practice, and Policy*. Advancing health equity and eliminating health disparities are and continue to be critical areas of great interest to the journal. Healthy People 2030 defines health equity as the attainment of the highest level of health for people: “Achieving health equity requires valuing everyone equally with focused and ongoing societal efforts to address avoidable inequities, historical and contemporary injustices, and social determinants of health —and to eliminate disparities in health and health care (1).” This new collection will feature articles that explore social determinants of health and the broad range of social, economic, political, and psychosocial factors that directly or indirectly shape health outcomes and contribute to health disparities. Health is not only the absence of disease but also the presence of the resources and supports that people need to thrive. This collection is expected to publish with 11 articles.

Our next scheduled collection will be *Sleep Deprivation, Sleep Disorders, and Chronic Disease*. Sleep is an essential daily behavior that supports physical, emotional, and psychological well-being (4,5). Nearly every body system depends on satisfactory sleep quality and quantity for routine healing, repair, and restoration (6). Sleep deprivation, poor sleep routines, adverse life events, and sleep disorders (eg, restless legs syndrome, insomnia) have a negative effect on health (6). In addition, particularly short sleep duration may be associated with chronic diseases (7,8). Emerging studies show how the COVID-19 pandemic significantly changed daily routines and affected mental health, including an increase among the general population in the prevalence of sleep disturbances during quarantine periods (9). This collection will include articles that bring increased attention to the relationship between sleep and chronic disease and will offer insight into the successes and challenges of public health strategies to improve sleep quality.

State and local health departments make up the national public health infrastructure (10,11), and we plan to publish a fall collection entitled *State and Local Health Departments: Research, Surveillance, and Evidence-Based Public Health Practices*. Recognizing the importance of this infrastructure, PCD will disseminate peer-reviewed content that highlights 1) public health practices across local and state health departments that strengthen their ability to respond to numerous health concerns, 2) the use of innovative tools and programs to help prevent and control chronic disease in the US, 3) current epidemiologic and surveillance activities, and 4) collaborations and partnerships to advance health equity and address persistent health disparities. We will feature work from numerous state and local health departments in this collection.

Our fifth collection is entitled *Advancing Chronic Disease Data Modernization Enhancements to Meet Current and Future Public Health Challenges*. The nation’s health data systems are often antiquated, resulting in myriad negative effects on chronic disease prevention and health promotion efforts (12). In addition, antiquated data management systems often become difficult to support, maintain, scale, or integrate into new platforms (12,13). Enhancing the nation’s data systems will require important changes to ensure skilled people are in place to manage them, new operational processes are adopted, and appropriate policies are established to facilitate monitoring and evaluation of data modernization action plans (13,14). This collection will address solutions to these challenges as well as examine evolving technologies that can transform the way public health data are collected and analyzed.

Evaluation is one of the 10 essential public health functions and is critical to effective public health practice, especially given its importance in monitoring and measuring the quality, pace, and direction of interventions and tracking their progress and impact (15). Our sixth collection, *Tools and Techniques to Effectively and Suitably Conduct Program Evaluation,* will include articles that provide instructional content to support professional development. This collection will focus on the “how-to” practical application of public health methods in performance monitoring and program evaluation.

And finally, our last collection of the year will be articles from our annual Student Paper Contest**.** This contest has been a huge hit with students (and their advisors), and we are pleased to include submissions from students at the high school, undergraduate, and graduate levels; recent postgraduates; and medical residents (16). Topics cover the full scope of the journal’s interest in the prevention, screening, and surveillance of chronic diseases, including but not limited to arthritis, asthma, cancer, depression, diabetes, obesity, cardiovascular disease, and COVID-19 and population-based interventions to address them. We have already posted the announcement for the 2024 Student Paper Contest, and students and interested advisors can find more information on our Announcements page (https://www.cdc.gov/pcd/announcements.htm).

We also are looking ahead to collections in 2024. In addition to the 7 collections scheduled for this year, we anticipate publishing a collection early in 2024 devoted to implementing and sustaining policy, systems, and environmental approaches to support healthy behaviors in diverse settings (see Current Call for Papers below and https://www.cdc.gov/pcd/announcements.htm).

## Current Calls for Papers

We currently have open calls for manuscript submissions in the following topic areas:

**Collection from authors in the National Center for Chronic Disease Prevention and Health Promotion (NCCDPHP) featuring PCD GIS Snapshots.** The journal remains committed to disseminating peer-reviewed content featuring work led by NCCDPHP divisions, partners, and collaborators. As part of this ongoing effort, we are offering a unique opportunity for divisions to submit papers generated by NCCDPHP authors for consideration for publication in a collection focused specifically on PCD’s GIS Snapshots articles. This collection will feature peer-reviewed GIS Snapshot papers that use maps to visually highlight chronic disease outcomes, risk factors, and relevant community characteristics, policies, and programs. This collection is scheduled for publication in the spring of 2024.

## Updates on Guidance to Authors: Health Equity Considerations

PCD continues to identify best practices and provide guidance to authors on how best to discuss them in papers submitted to the journal for consideration. As part of that commitment, we recently posted “Health Equity Considerations” on our website (https://www.cdc.gov/pcd/for_authors/types_of_articles.htm). This section offers guidance for authors when conducting and summarizing findings from research, evaluation, and practice. Authors are encouraged to consider the following 5 questions when drafting a paper intended for the journal:

1. How do you define health equity as it applies to the work discussed in the manuscript?
2. How were aspects of advancing health equity applied when identifying key partners?
3. How were aspects of advancing health equity used in designing and implementing public health approaches to improve health?
4. How were aspects of health equity used when conducting the evaluation or research described in the manuscript?
5. How was the health equity lens used to identify future recommendations on ways to advance health equity with regard to a) identifying partnerships, b) designing and implementing public health approaches, and/or c) conducting evaluation or research?

We will continue to post guidance related to principles and practices in health equity and include updates on our website.

## PCD Recognized as a National Resource on the Integration of Diversity, Equity, and Inclusion (DEI) in Scholarly Publishing

### PCD Demonstrates National Leadership

I have been fortunate in my position as PCD’s editor in chief to be asked to participate in ongoing discussions and resolutions around diversity, equity, and inclusion in scholarly publishing. I am encouraged to see this work going on in our community and to participate in that work. In particular, I was active in several initiatives from the Council of Science Editors (CSE), contributing to articles on ways to integrate DEI in scholarly publishing (https://www.cdc.gov/pcd/issues/2023/23_0051.htm) and updating CSE’s style manual and its Recommendations for Promoting Integrity in Scientific Journal Publications. In addition, I served as an instructor in a DEI short course at CSE’s annual meeting, providing an editor in chief’s perspective on addressing DEI; discussing how to report race, ethnicity, and gender in publications; and use of inclusive language in scientific publishing. CSE also conducted the CSE Ethics Clinic that focused on DEI case studies, which I conceptualized, and I was excited to be a presenter and engage with others on these cases.

It has been a rewarding experience to participate in the changes happening in scholarly publishing and to ensure that our journal is at the forefront of these changes. We will continue to be involved in efforts to promote industry standards, best practices, and “how to” approaches that result in the adoption of day-to-day operational policies that improve the criticality, trustworthiness, and transferability of DEI best practices in the field.

### Optional Self-Reported Demographic Data: Preliminary Findings

PCD is committed to fostering a scientific community that supports and benefits from the talents of researchers from a wide range of backgrounds. As part of this commitment, we have initiated optional self-reporting of demographic data through our ScholarOne manuscript tracking system. Preliminary data were aggregated and anonymized before being analyzed and reported to us. ScholarOne provided demographic data collected from August 2022 through April 2023. 

**Figure 1:**
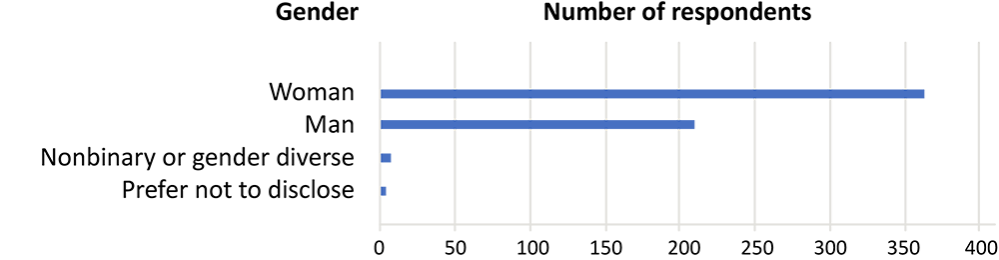
Self-reported gender of manuscript management system users, *Preventing Chronic Disease*, April 2023.

As of August 2022, a total of 2,121 prospective authors or “users” had submitted manuscripts to PCD for consideration for publication. Of these, 591 (27.9%) users provided optional self-reported data regarding gender, race, and ethnicity. Women accounted for 363 respondents (61%), 210 (35.5%) were male, 11 (1.9%) preferred not to disclose, and 7 (1.3%) identified as nonbinary or gender diverse (Figure 1). 

**Table:** 

Gender	Number of respondents
Woman	363
Man	210
Nonbinary or gender diverse	7
Prefer not to disclose	11

**Figure 2:**
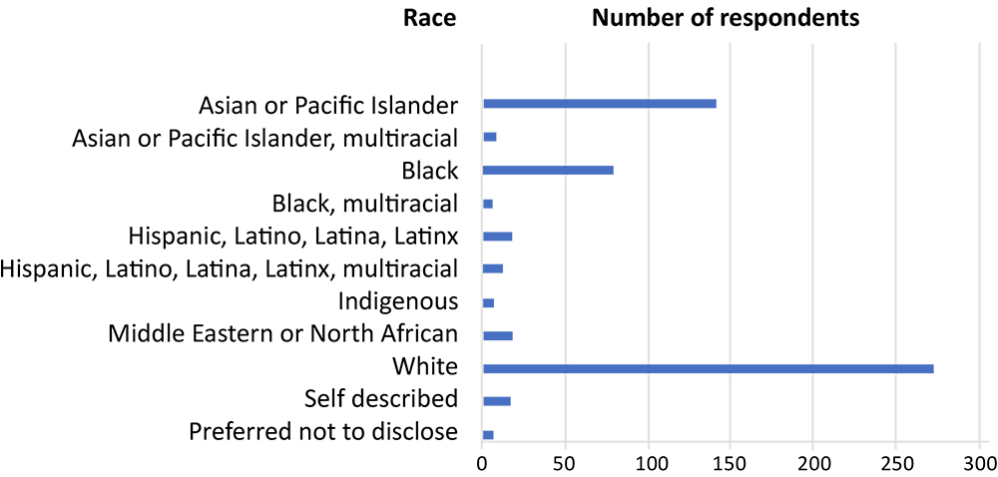
Self-reported race of manuscript management system users, *Preventing Chronic Disease*, April 2023. Note that labels do not correspond to federally designated race categories and are a function of the Clarivate data retrieval system, which reports subcategories of race. The indigenous category includes North American Indian, South American Indian, and Aboriginal or Torres Strait Islander. Source: Clarivate Analytics/ScholarOne (www.Clarivate.com).

A diverse distribution of our users reported race as follows (percentages are rounded and do not add to 100%): Asian or Pacific Islander, 141 (23.9%); Asian or Pacific Islander, multiracial, 9 (1.5%); Black, 79 (13.4%); Black, multiracial, 7 1.2%); Hispanic or Latino, Latina, Latinx, 19 (3.2%); Indigenous (includes North American Indian, South American Indian, Aboriginal or Torres Strait Islander), 7 (1.2% ); Middle Eastern or North African, 19 (3.2%); preferred not to disclose 18 (3.0%); White, 272 (46%); and self-described 7 (1.2%) (Figure 2).

**Table:** 

Race	Number of respondents
Asian or Pacific Islander	141
Asian or Pacific Islander, multiracial	9
Black	79
Black, multiracial	7
Hispanic, Latino, Latina, Latinx	19
Hispanic, Latino, Latina, Latinx, multiracial	13
Indigenous	7
Middle Eastern or North African	19
White	272
Self described	7
Preferred not to disclose	18

**Figure 3:**
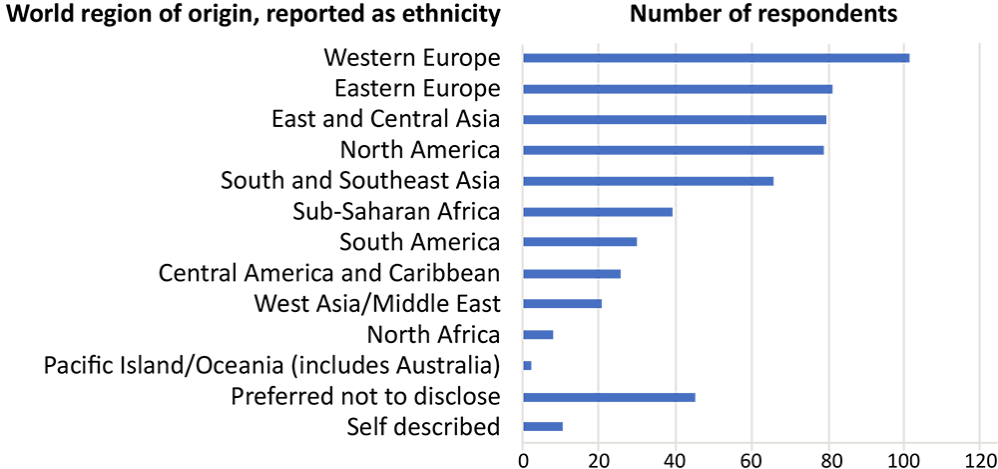
Self-reported world region of origin of manuscript management system users queried about ethnicity, *Preventing Chronic Disease*, April 2023. Source: Clarivate Analytics/ScholarOne (www.Clarivate.com).

The 591 PCD users who self-reported represented a broad range of ethnicities, which our database reported by world region. In rank order, origins were Western Europe, 102 (17.2%); Eastern Europe, 83 (14.0%); East and Central Asia, 79 (13.4%); North America, 77 (13.0%); South and Southeast Asia, 66 (11.2%); preferred not to disclose, 46 (7.8%); Sub-Saharan Africa, 39 (6.6%); South America, 30 (5.1%); Central America and Caribbean, 26 (4.4%); West Asia/Middle East, 21 (3.6%); self-described, 11 (1.9%); North Africa, 8 (1.4%); and Pacific Islands/Oceania (includes Australia) 3 (0.5%) (Figure 3).

**Table:** 

World region of origin, reported as ethnicity	Number of respondents
Western Europe	102
Eastern Europe	83
East and Central Asia	79
North America	77
South and Southeast Asia	66
Sub-Saharan Africa	39
South America	30
Central America and Caribbean	26
West Asia/Middle East	21
North Africa	8
Pacific Island/Oceania(includes Australia)	3
Preferred not to disclose	46
Self described	11

We realize that these findings regarding gender, race, and ethnicity are preliminary and represent only 28% of the total number of authors who have used our manuscript submission system. Our leadership will continue to encourage users to accept the invitation to voluntarily provide information regarding gender, race, and ethnicity. We will continue to use this information to guide our efforts to engage the diverse groups of individuals who serve on our volunteer groups, submit papers to the journal for consideration, serve as peer reviewers, and are appointed as guest editors. 

## Conclusion

In many ways, PCD serves as a leader in providing support to its authors before they submit papers for consideration. Special thanks go to our volunteer groups who bring a wealth of training and expertise to the journal. Prospective authors are encouraged to sign up to receive ongoing updates about the journal’s innovations and to visit the journal’s website for current announcements. Should you be interested in submitting a proposal to generate a collection, see our instructions page for detailed guidance (https://www.cdc.gov/pcd/collections/collection_submission_instructions.htm). The journal has certainly evolved over the years, and our commitment to excellence has never wavered. We hope that our PCD readers continue to find us committed to constantly thinking about ways to improve our day-to-day operations.

## Appendix Guest Editorial Board for PCD Collection, Combating Racism Through Research, Training, Practice, and Public Health Policies

### Guest Editorial Board Co-Chairs

**Table:**

	**Jeffrey E. Hall, PhD, MA, MSPH, CPH (Co-Chair), **Deputy Director, Office of Minority Health and Health Equity and Chief of the Minority Health and Health Equity Team, Office of Minority Health and Health Equity, Centers for Disease Control and Prevention
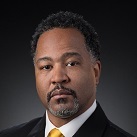
	**L. Ebony Boulware, MD, MPH (Co-Chair), **Dean, Wake Forest University School of Medicine, and Vice Chief Academic Officer and Chief Science Officer, Advocate Health
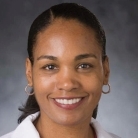

### Guest Editorial Board Members

**Table:**

	**Sergio Aguilar-Gaxiola, MD, PhD,** Professor of Clinical Internal Medicine, School of Medicine, University of California, Davis
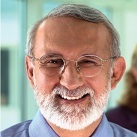
	**Tabia Henry Akintobi, PhD, MPH,** Professor, Community Health and Preventive Medicine; Associate Dean, Community Engagement; Director, Prevention Research Center, Morehouse School of Medicine
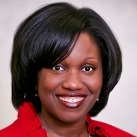
	**Philip M. Alberti, PhD, **Founding Director, AAMC Center for Health Justice and Senior Director, Health Equity Research and Policy, Association of American Medical Colleges
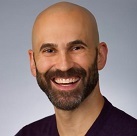
	**Brian Armour, PhD, **Associate Director for Science, Office of Smoking and Health, Centers for Disease Control and Prevention
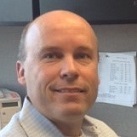
	**David Chae, ScD, MA, **Associate Professor; Associate Dean for Research; Director, Society, Health and Racial Equity (SHARE) Lab, School of Public Health and Tropical Medicine, Tulane University
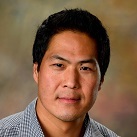
	**Fátima Coronado, MD, MPH, **Associate Director for Science, Division for Heart Disease and Stroke Prevention, National Center for Chronic Disease and Health Promotion, Centers for Disease Control and Prevention
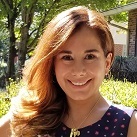
	**Shanna N. Cox, MSPH, **Associate Director of Science, Division of Reproductive Health, National Center for Chronic Disease Prevention and Health Promotion, Centers for Disease Control and Prevention
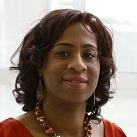
	**Sheba M. George, PhD, **Associate Professor; Director, CDU Community Health Worker Academy; Program Director, Health Careers Opportunity Program, Department of Preventive and Social Medicine, Charles R. Drew University of Medicine and Science
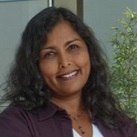
	**Latha Palaniappan, MD, MS, **Professor of Medicine, Primary Care and Population Health, Stanford University School of Medicine
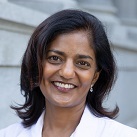
	**Austin Porter III, DrPH, MPH, **Deputy Chief Science Officer, Arkansas Department of Health; Assistant Professor, Health Policy and Management, Fay W. Boozman College of Public Health; University of Arkansas for Medical Sciences
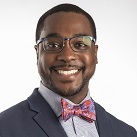
	**Michael L. Sells, PhD, MS, CHES, **Public Health Advisor, Advancing Population Health Team, Program Development and Services Branch, Division of Heart Disease and Stroke Prevention, National Center for Chronic Disease Prevention and Health Promotion, Centers for Disease Control and Prevention
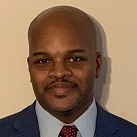
	**Judith Lee Smith, PhD, **Senior Behavioral Scientist, Lead, Behavioral and Applied Research Team, Epidemiology and Applied Research Branch, Division of Cancer Prevention and Control, National Center for Chronic Disease Prevention and Health Promotion, Centers for Disease Control and Prevention
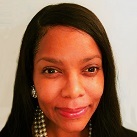
	**Evelyn Twentyman, MD, MPH, **Medical Epidemiologist, Office of Medicine and Science, National Center for Chronic Disease Prevention and Health Promotion, Centers for Disease Control and Prevention
